# Socioeconomic Disparities in Preemptive Kidney Transplant Rates in Children

**DOI:** 10.34067/KID.0000000802

**Published:** 2025-04-07

**Authors:** Sarah Kizilbash, Chung-II Wi, Madison Roy, Warren T. McKinney, Sandra Amaral, Samy Riad, Young Juhn

**Affiliations:** 1 Department of Pediatrics, University of Minnesota, Minneapolis, Minnesota; 2 Precision Population Science Lab and Department of Pediatric and Adolescent Medicine, Mayo Clinic, Rochester, Minnesota; 3 Precision Population Science Lab and Department of Quantitative Health Science, Mayo Clinic, Rochester, Minnesota; 4 Division of Nephrology, Hennepin Healthcare Research Institute, Minneapolis, Minnesota; 5 Department of Medicine, Hennepin Healthcare, Minneapolis, Minnesota; 6 Department of Pediatrics and Epidemiology, Children's Hospital of Philadelphia, University of Pennsylvania Perelman School of Medicine, Philadelphia, PA; 7 Division of Nephrology and Hypertension, Department of Internal Medicine, Mayo Clinic, Rochester, Minnesota; 8 Precision Population Science Lab and Department of Pediatric and Adolescent Medicine/Internal Medicine, Mayo Clinic, Mayo Clinic Health System, Rochester, Minnesota

**Keywords:** kidney transplantation

## Abstract

**Key Points:**

- Pediatric preemptive kidney transplantation rates in the United States vary by socioeconomic status (SES).
- The HOUsing-based index of SES, an individual-level SES measure derived from residential addresses, effectively predicts pediatric preemptive kidney transplant rates.

**Background:**

Preemptive kidney transplantation in children is associated with better patient and graft survival compared with transplants after dialysis. However, preemptive kidney transplantation rates in children remain low. This study aimed to evaluate the effect of socioeconomic status (SES) on preemptive transplantation in children.

**Methods:**

Our study included 173 Minnesota-resident pediatric kidney transplant recipients (younger than 18 years) transplanted at the University of Minnesota from 2010 to 2020. Using the HOUsing-based index of SES (HOUSES) index, a validated, individual SES measure based on housing units categorized into quartiles (Q1: lower SES; Q2–Q4: higher SES), we applied mixed-effects multivariable logistic models to examine the effects of HOUSES on preemptive kidney transplants and pretransplant dialysis duration.

**Results:**

Of 173 pediatric kidney transplant recipients, 46 (26.6%) received a preemptive transplant, and of 109 recipients with dialysis duration data, 39 (35.8%) received dialysis for >1 year. After adjusting for age at kidney failure, sex, donor type, insurance type, and underlying cause of kidney failure, we observed significantly lower odds of preemptive transplantation among Q1 recipients compared with Q2–4 recipients (adjusted odds ratio, 0.31; 95% confidence interval, 0.11 to 0.90; *P* = 0.03).

**Conclusions:**

Using the HOUSES index, we found significant socioeconomic disparities in preemptive kidney transplantation rates among children.

## Visual Abstract

**Figure:**
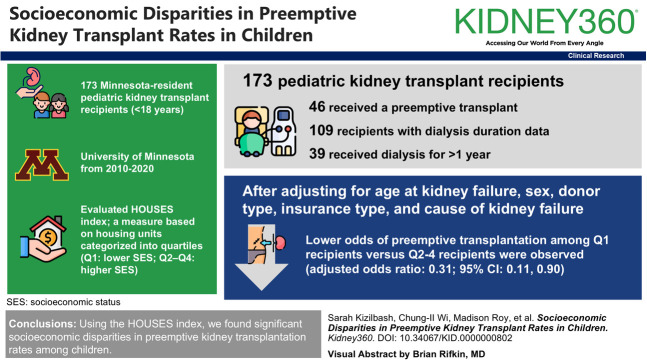

## Introduction

Kidney transplantation is the treatment of choice for children with ESKD as it is associated with survival benefits, improved growth, better neurocognitive development, and a higher quality of life compared with chronic dialysis.^1–5^ Children who undergo transplants before requiring dialysis (preemptive transplantation) demonstrate better patient and graft survival compared with those exposed to pretransplant dialysis.^6^ Preemptive transplants are also associated with superior neurocognitive development compared with dialysis exposure of >3 months.^7^ The 2020 Kidney Disease Improving Global Outcomes Clinical Practice Guidelines recommend preemptive transplantation for all children with advanced CKD.^8^ However, only 24.2% of children with incident ESKD received a preemptive transplant in the United States in 2021.^9^ Identifying factors associated with poor preemptive transplantation rates in children is necessary to inform interventions intended to improve rates.

Social deprivation, public insurance, minority race and ethnicity, and low educational attainment are associated with a lower likelihood of a preemptive transplant and a higher likelihood of longer pretransplant dialysis exposure.^10–12^ Pediatric data on the effects of socioeconomic factors on preemptive transplantation are sparse and conflicting. A retrospective study of 768 children using data from the Australia and New Zealand Dialysis and Transplant Registry (1993–2012) found no association between socioeconomic status (SES) quintiles and access to preemptive kidney transplantation.^13^ Conversely, a United Kingdom Renal Registry study of 2160 children and a French study of 1115 children demonstrated fewer preemptive transplants in children from socially deprived neighborhoods.^14,15^ These studies are limited by SES measures, employing area-level measures derived from zip/postcodes and census data. Area-level SES measures may inaccurately capture individual-level SES, leading to SES misclassification and misleading results.^16^

To overcome the limitations of the existing SES measures, we used a novel SES measure, the HOUsing-based index of SES (HOUSES), to determine the association between individual-level SES and preemptive kidney transplantation among pediatric kidney transplant recipients. HOUSES is an individual-level SES measure generated by linking address information from electronic health records or other data sources to publicly available real property data. It captures the household's net wealth and income, social and environmental resources, and the effects of the building features on health.^17^ We hypothesized that fewer children with lower SES, defined by the HOUSES quartiles, would access preemptive transplants. We also hypothesized a longer pretransplant dialysis duration among nonpreemptive pediatric kidney transplant recipients with lower SES. This study is important as it evaluates the effect of individual-level SES, measured by HOUSES, on preemptive kidney transplant rates and pretransplant dialysis duration, providing insights that could inform policies.

## Methods

### Study Design

This retrospective cohort study evaluated the differential effect of individual-level SES on preemptive kidney transplantation and pretransplant dialysis duration among pediatric kidney transplant recipients. The Institutional Review Boards at the University of Minnesota and the Mayo Clinic approved the study. The clinical and research activities being reported are consistent with the Principles of the Declaration of Istanbul as outlined in the “Declaration of Istanbul on Organ Trafficking and Transplant Tourism.”

### Study Population

Using a prospectively maintained, Institutional Review Board-approved, solid organ transplant database at the University of Minnesota, we identified all pediatric kidney transplant recipients (age younger than 18 years at the time of transplant) who were transplanted at the University of Minnesota between January 1, 2010, and January 1, 2020, and were residents of Minnesota at the time of their transplant. The transplant database contains post-transplant, peritransplant, and minimal pretransplant data on recipients and donors. Information on demographics, pretransplant comorbidities, transplant surgery variables, post-transplant complications, and outcomes are captured through manual chart abstraction and electronic data transfer from the electronic medical record.

**Figure 1:**
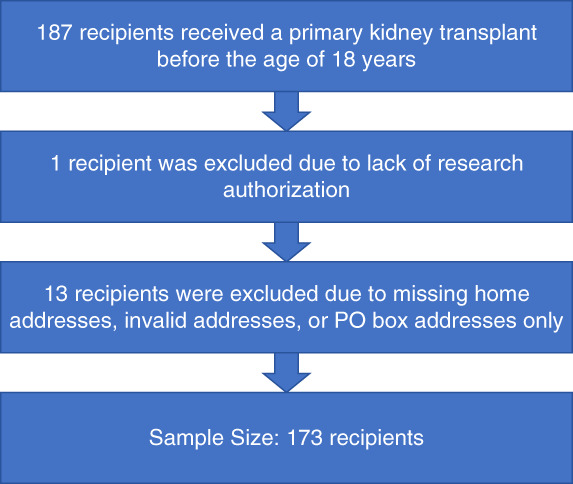
**Flow diagram of participant inclusion and exclusion.** PO, post office.

Of the 187 unique potential participants, one was excluded for lack of research authorization, and 13 were excluded due to missing home addresses, invalid addresses, or post office box addresses only. For patients with multiple transplants, the first kidney transplant was included (Figure 1).

### Study Variables

Our study included the following variables: age at ESKD, age at transplant, cause of ESKD, sex, race, donor source, prior transplant, insurance type, recipient's body mass index, and pretransplant dialysis duration.

Using the recipient's residential addresses, we computed each recipient's HOUSES score on the basis of the County's publicly available property data for individual housing units.

### Study Exposure

Our primary exposure of interest was the HOUSES index at the time of transplant. We used housing data to determine the individual-level SES of all pediatric recipients at the time of transplant. HOUSES is a robust, individual-level SES measure computed from four variables (number of bedrooms, number of bathrooms, square footage of the unit, and estimated building value of the unit) ascertained from the County Assessor's office based on home addresses, which is normalized within each county.^17^ Each property item corresponding to an individual's address is standardized into a z-score and aggregated into an overall z-score for the four items such that a higher HOUSES score indicates higher SES. Although ownership was one of the 14 variables initially considered in the original HOUSES study, it was not included in the final index alongside the four selected variables, as adding ownership to the model did not result in significantly different outcomes compared with the parsimonious model based on four variables only. The HOUSES index was divided into quartiles (Q1 indicating the lowest SES). Construct validity of HOUSES has been assessed extensively using health outcomes with established association with SES, as presented in Supplemental Tables 1 and 2. To make HOUSES scalable, an automated cloud-based platform was recently established, making the HOUSES formulation process automatic and making HOUSES available to nationwide users. The HOUSES data are updated annually as property information is refreshed. Therefore, the HOUSES value for a given person in 2010 may differ from that in 2020, depending on changes in their address and/or property assessment. For the purposes of our study, we used the HOUSES data at the time of transplantation.

### Study Outcomes

Our primary outcome of interest was preemptive transplantation (defined as transplant before dialysis). At our institution, we refer children for transplant evaluation at a GFR of 20–30 ml/min per 1.73 m^2^. After evaluation completion, patients are listed in an “inactive status” on the deceased donor waitlist. In accordance with Kidney Disease Improving Global Outcomes guidelines, we proceed with a preemptive living donor transplant (if a living donor is available) or activate the child on the deceased donor waitlist for a preemptive deceased donor transplant when the GFR falls below 15 ml/min per 1.73 m^2^ or if the patient becomes significantly symptomatic even at a GFR >15 ml/min per 1.73 m^2^.

The secondary outcome included dichotomized pretransplant dialysis duration (<1 versus ≥1 year). We defined dialysis duration as the interval between the first chronic dialysis session and kidney transplant and categorized it into <1 year duration and ≥1 year duration.

### Statistical Analysis

Descriptive statistics were used to summarize the characteristics of the cohort by dichotomous HOUSES quartiles (Q1 versus Q2–4). This approach is consistent with our earlier study, which demonstrated an association between HOUSES and the risk of graft loss in patients residing in Olmsted County, Minnesota.^18^ The consolidation of the Q2–4 quartiles also allowed us to conserve power for statistical analysis, given our small sample size. We used ANOVA and t-tests for continuous variables and Pearson chi-squared test and Fisher exact tests for categorical variables to measure the association between HOUSES categories and patient characteristics at the time of the transplant. We used mixed effects multivariable logistic models (county as a random effect) to evaluate the effect of HOUSES on preemptive transplantation rates and pretransplant dialysis duration (<1 versus ≥1 year). The random effect of the county was used because the HOUSES index provides a relative ranking of SES within a county. This approach accounts for outcome variations across counties while preserving the within-county SES ranking. We selected covariates for inclusion in the multivariable model on the basis of their association with the outcome of interest, indicated by a *P* value of <0.2 on univariate analysis.

Since access to preemptive transplants may vary by donor type, we conducted stratified subanalyses using separate models for living and deceased donor recipients. We used a mixed effects multivariable logistic model (county as a random effect) to evaluate the effect of HOUSES on preemptive transplantation rates in living donor recipients. Similarly, we used a mixed effects multivariable logistic model (county as a random effect) to evaluate the effect of HOUSES on preemptive transplantation rates in deceased donor recipients. We selected covariates for inclusion in the multivariable model based on their association with the outcome of interest, indicated by a *P* value of <0.2 on univariate analysis or variables previously reported in the literature as potential confounders.^19^ However, we could not include ESKD cause as a variable in the analysis stratified by donor source because no living donor recipients with GN underwent a preemptive transplant. This absence of data resulted in complete separation, preventing outcome estimation based on the cause of ESKD.

Since many centers do not consider candidates younger than 2 years for kidney transplantation, we also conducted sensitivity analyses restricting the study population to those younger than 2 years at the onset of ESKD. We used mixed effects multivariable logistic models (county as a random effect) to evaluate the effect of HOUSES on preemptive transplantation rates and pretransplant dialysis duration (<1 versus ≥1 year) in children older than 2 years at the onset of ESKD. We selected covariates for inclusion in the multivariable model on the basis of their association with the outcome of interest, indicated by a *P* value of <0.2 on univariate analysis.

All analyses were performed using SAS v9.4 software (SAS Institute, Cary, NC).

## Results

### Baseline and Demographic Characteristics

Our final analysis cohort included 173 pediatric kidney transplant recipients.

Table 1 presents the baseline characteristics of the study population by the dichotomous HOUSES score. Overall, the median age at ESKD was 11.4 years (range, 0.01–17.9), and the median age at transplant was 12.2 years (range, 0.77–19.6). Of the recipients, 54.9%% were male, and 72.8% were White. Preemptive transplantation was observed in 46 (26.6%) recipients, while pretransplant dialysis in 127 (73.4%) recipients. Compared with Q2–4, Q1 recipients were less likely to have private insurance (43.2% versus 65.9%; *P* = 0.008). We found no differences in donor source, HLA mismatch, or recipient's body mass index at the time of transplant between Q1 and Q2–4 recipients (Table 1).

**Table 1:** Characteristics of the study cohort by HOUsing-based index of Socio-Economic Status

Variables	Q2–Q4 (*n*=−129)	Q1 (*n*=44)	*P* Value
**Age at ESKD**			0.64^a^
Median	11.4	11.6	
Range	0.01–17.9	0.01–17.9	
**Age at transplant**			0.33^a^
Median	12.2	12.5	
Range	0.77–18.5	1.8–19.6	
**Sex, *n* (%)**			0.52^b^
Female	60 (46.5)	18 (40.9)	
Male	69 (53.5)	26 (59.1)	
**Race, *n* (%)**			0.24
American Indian or Alaskan Native	7 (5.4)	3 (6.8)	
Asian	13 (10.1)	8 (18.2)	
Black	10 (7.8)	6 (13.6)	
White	99 (76.7)	27 (61.4)	
**Ethnicity, *n* (%)**			0.74^b^
N-Miss	9	0	
Non-Hispanic White	69 (57.5)	24 (54.5)	
Other	51 (42.5)	20 (45.5)	
**Insurance, *n* (%)**			0.008^b^
Private	85 (65.9)	19 (43.2)	
Public/other	44 (34.1)	25 (56.8)	
**Donor type, *n* (%)**			0.19^b^
Deceased	50 (38.8)	22 (50.0)	
Living	79 (61.2)	22 (50.0)	
**HLA mismatch**			0.38^a^
N-Miss	7	0	
Median	4.0	4.0	
Range	0.0–6.0	0.0–6.0	
**Recipient BMI (prior to transplant)**			0.39^a^
N-Miss	17	5	
Median	18.8	17.9	
Range	13.8–36.7	12.2–36.9	
**Cause of ESKD, *n* (%)**			0.69^b^
CAKUT	64 (49.6)	18 (40.9)	
FSGS	22 (17.1)	6 (13.6)	
GN	5 (3.9)	2 (4.5)	
Other	31 (24.0)	14 (31.8)	
Unknown	7 (5.4)	4 (9.1)	

BMI, body mass index; CAKUT, congenital anomalies of the kidney and urinary tract; N-miss, missing.

a Linear Model ANOVA.

b Pearson chi-squared test.

### Preemptive Transplantation

Compared with Q2–4, Q1 recipients were less likely to have undergone a preemptive transplant (13.6% versus 31.0%; *P* = 0.02). The preemptive transplant group included a preponderance of male recipients (preemptive versus nonpreemptive: 71.7% versus 48.8%; *P* = 0.007), privately insured recipients (preemptive versus nonpreemptive: 78.3% versus 53.5%; *P* = 0.003), and Q2–4 recipients (preemptive versus nonpreemptive: 87.0% versus 70.1%; *P* = 0.024) compared with the nonpreemptive group. We also found differences in preemptive versus nonpreemptive transplant rates by the cause of ESKD (*P* = 0.004), observing higher rates of preemptive transplants in patients with congenital anomalies of the kidney and urinary tract (preemptive versus nonpreemptive: 67.4% versus 40.2%) and lower rates of preemptive transplants in patients with FSGS (preemptive versus nonpreemptive: 2.2% versus 21.3%; Supplemental Table 3).

**Figure 2:**
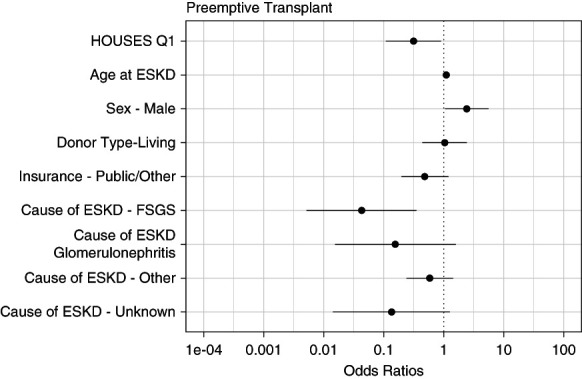
**Predictors of preemptive kidney transplantation in children.** HOUSES, HOUsing-based index of Socio-Economic Status.

After adjusting for age at ESKD, sex, donor type, insurance type, and the cause of ESKD, we observed significantly lower odds of preemptive transplantation for Q1 recipients compared with Q2–4 recipients (adjusted odds ratio [aOR], 0.31; 95% confidence interval [CI], 0.11 to 0.90; *P* = 0.03; Table 2). Furthermore, we found that age at ESKD, sex, and the cause of ESKD were significant predictors of preemptive transplantation (Figure 2).

**Table 2:** Preemptive transplantation—multivariable model

Variables	OR	95% CI	*P* Value
**HOUSES**			
Q2–4	Ref		0.03
Q1	0.31	0.11 to 0.90	
Age at ESKD	1.1	1.02 to 1.2	0.008
**Sex**			
Female	Ref		0.04
Male	2.42	1.04 to 5.6	
**Donor type**			
Deceased	Ref		0.94
Living	1.03	0.44 to 2.4	
**Insurance type**			
Private	Ref		0.11
Public/other	0.48	0.19 to 1.2	
**Cause of ESKD**			
CAKUT	Ref		
FSGS	0.04	0.005 to 0.35	0.003
GN	0.16	0.02 to 1.6	0.12
Other	0.59	0.24 to 1.4	0.24
Unknown	0.14	0.01 to 1.3	0.08

CAKUT, congenital anomalies of the kidney and urinary tract; CI, confidence interval; HOUSES, HOUsing-based index of Socio-Economic Status; OR, odds ratio.

### Stratification by Donor Source

When stratified by donor source, living-donor recipients in the Q1 group had significantly lower odds of receiving a preemptive transplant compared with those in the Q2–4 group after adjusting for age at transplant, sex, race, and insurance type (aOR, 0.23; 95% CI, 0.05 to 0.98; *P* = 0.046). However, we observed no significant difference in the odds of preemptive transplantation in Q1 versus Q2–4 groups among the deceased donor recipients (aOR, 0.50; 95% CI, 0.12 to 2.1; *P* = 0.35; Table 3).

**Table 3:** Preemptive transplant—stratified analysis by donor type

Variables	OR	95% CI	*P* Value
**Living donor recipients**			
HOUSES			0.046
*Q2–4*	Ref		
*Q1*	0.23	0.05 to 0.98	
Age at ESKD	1.07	0.98 to 1.17	0.12
Sex			0.002
*Female*	Ref		
*Male*	5.88	1.93 to 17.89	
Race	1.74	0.56 to 5.46	0.34
*Non-Hispanic White*			
*Other*			
Insurance type	0.35	0.09 to 1.28	0.11
*Private*			
*Public/other*			
**Deceased donor recipients**			
HOUSES	0.50	0.12 to 2.14	0.35
*Q2–4*			
*Q1*			
Age at ESKD	1.002	0.89 to 1.12	0.96
Sex	1.49	0.43 to 5.20	0.53
*Female*			
*Male*			
Race	0.59	0.17 to 2.15	0.43
*Non-Hispanic White*			
*Other*			
Insurance type	0.51	0.14 to 1.84	0.30
*Private*			
*Public/other*			

CI, confidence interval; HOUSES, HOUsing-based index of Socio-Economic Status; OR, odds ratio.

### Pretransplant Dialysis Duration

A higher proportion of Q1 recipients receiving pretransplant dialysis for >1 year (47.2% versus 30.1%; *P* = 0.08) compared with Q2–4 recipients; however, the difference did not achieve statistical significance. Recipients with <1 year of pretransplant dialysis were more likely to be older at the onset of ESKD (median age at ESKD in years: 12.3 versus 9.6; *P* = 0.024). Furthermore, recipients with <1 year of pretransplant dialysis were more likely to be non-Hispanic White (68.3% versus 35.1%; *P* = 0.001), recipients of living donors (65.1% versus 21.6%; *P* < 0.001), and privately insured (68.3% versus 32.4%; *P* < 0.001; Supplemental Table 4).

**Figure 3:**
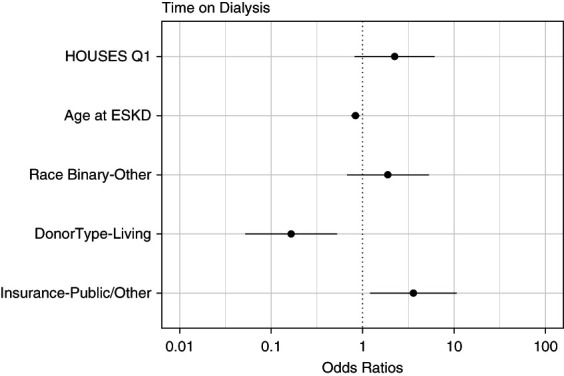
Predictors of pretransplant dialysis duration of ≥1 year.

After adjusting for age at ESKD, race, donor type, and insurance type, we observed no significant effect of the HOUSES index on pretransplant dialysis duration. However, the age at ESKD, donor type, and insurance type were significant predictors of pretransplant dialysis duration (Figure 3 and Table 4).

**Table 4:** Pretransplant dialysis duration (<1 versus ≥1 year)—multivariable model

Variables	OR	95% CI	*P* Value
**HOUSES**			
Q2–4	Ref		0.12
Q1	2.3	0.82 to 6.2	
Age at ESKD	0.84	0.76 to 0.93	<0.001
**Race**			
Non-Hispanic White	Ref		
Other	1.9	0.67 to 5.3	0.24
**Donor type**			
Deceased	Ref		
Living	0.17	0.05 to 0.52	0.002
**Insurance type**			
Private	Ref		
Public/other	3.6	1.2 to 10.7	0.02

CI, confidence interval; HOUSES, HOUsing-based index of Socio-Economic Status; OR, odds ratio.

### Sensitivity Analysis

When the study cohort was restricted to patients who developed ESKD after the age of 2 years, Q1 recipients remained significantly less likely to receive a preemptive kidney transplant compared with Q2–4 recipients after adjusting for age at ESKD, sex, donor type, insurance type, and the cause of ESKD (aOR, 0.28; 95% CI, 0.09 to 0.80; *P* = 0.017; Table 5).

**Table 5:** Sensitivity analysis (age at ESKD onset >2 years)—preemptive transplant

Variables	OR	95% CI	*P* Value
**HOUSES**			0.02
Q2–4	Ref		
Q1	0.28	0.09 to 0.79	
Age at ESKD	0.98	0.89 to 1.08	0.66
**Sex**			0.03
Female	Ref		
Male	2.62	1.08 to 6.34	
**Donor type**			0.99
Deceased	Ref		
Living	1.00	0.40 to 2.49	
**Insurance type**			0.08
Private	Ref		
Public/other	0.41	0.16 to 1.09	
**Cause of ESKD**			0.003
CAKUT	Ref		
FSGS	0.04	0.005 to 0.33	
GN	0.15	0.02 to 1.55	0.11
Other	0.57	0.22 to 1.46	0.24
Unknown	0.18	0.02 to 1.77	0.14

CAKUT, congenital anomalies of the kidney and urinary tract; CI, confidence interval; HOUSES, HOUsing-based index of Socio-Economic Status; OR, odds ratio.

In the cohort restricted to patients older than 2 years at the onset of ESKD, the association between the HOUSES index and pretransplant dialysis duration approached statistical significance after adjusting for age at ESKD, race, donor type, and insurance type (aOR, 3.0; 95% CI, 0.91 to 9.8; *P* = 0.069; Table 6).

**Table 6:** Sensitivity analysis (age at ESKD onset >2 years)—pretransplant dialysis duration (<1 versus ≥1 year)

Variables	OR	95% CI	*P* Value
**HOUSES**			0.07
Q2–4	Ref		
Q1	2.99	0.91 to 9.83	
Age at ESKD	0.86	0.74 to 1.003	0.05
**Race**			0.03
Non-Hispanic White	Ref		
Other	3.67	1.09 to 12.30	
**Donor type**			0.002
Deceased	Ref		
Living	0.11	0.02 to 0.44	
**Insurance type**			0.09
Private	Ref		
Public/other	2.89	0.86 to 9.75	

CI, confidence interval; HOUSES, HOUsing-based index of Socio-Economic Status; OR, odds ratio.

## Discussion

This is the first study to examine the association between the HOUSES index and preemptive kidney transplantation in children. Using HOUSES as an individual-level SES measure, we found significant socioeconomic disparities in preemptive kidney transplantation rates in children, independent of age at ESKD, sex, donor type, insurance type, and the cause of ESKD. Furthermore, the disparities persisted among living donor recipients when the analysis was stratified by donor source. The association between the HOUSES index and the dialysis duration of ≥1 year also approached statistical significance for children who developed ESKD after the first 2 years of life. Our findings align with our previous adult study, showing lower rates of preemptive transplantation in patients with the lowest HOUSES quartile (Q1).^18^ This study highlights socioeconomic disparities in preemptive transplantation in children and demonstrates the utility of the HOUSES Index in identifying children who are at an increased risk of pretransplant dialysis exposure.

Previous studies evaluating the effects of SES on preemptive transplantation in children provide conflicting results. In a retrospective study of 8053 children with ESKD, Patzer *et al.*, using data from the United States Renal Data System, found no significant association between neighborhood poverty level and deceased donor preemptive kidney transplantation.^19^ Contrarily, a CKD in Children Study including 184 children with ESKD found a lower likelihood of a preemptive transplant in children with lower neighborhood income and higher neighborhood deprivation; however, these associations lost statistical significance after controlling for participant characteristics.^20^ Similarly, an Australia and New Zealand Dialysis and Transplant Registry study including 768 children (1993–2012) revealed no association between SES and preemptive kidney transplantation.^13^ Conversely, studies from the United Kingdom and France, including 2160 and 1115 children, respectively, found children from socially deprived neighborhoods to be less likely to receive a preemptive transplant.^14,15^ The inconsistent findings of previous studies might have resulted from using area-level measures of SES as proxies for individual-level SES. Registry studies frequently rely on area-level SES measures, as individual-level SES measures are not available in large datasets. However, area-level measures often yield inconsistent results due to imprecision and misclassification bias.^18^ Expanding national data collection to include individual-level measures, such as HOUSES, is important to examine the nuanced effects of SES on health care.

Of the 46 preemptive transplants in our study cohort, one third were deceased donor transplants and two thirds were living donor transplants. The relatively high proportion of deceased donor preemptive transplants in our cohort likely reflects our proactive approach to listing and activating patients on the deceased donor waitlist before their need for dialysis. Our stratified analysis revealed persistent SES disparities in living preemptive recipients, but none in deceased donor recipients. The absence of association between SES and preemptive deceased donor transplants might have resulted from our small sample size. However, SES disparities in living donor but none in deceased donor transplants mimic racial disparities seen in living donor preemptive transplants but not in deceased donor transplants.^19^ The characteristics of living donors—whether parents, siblings, or unrelated individuals—can significantly influence the dynamics of preemptive transplants. Parents, who are often the primary living donors for children, may face financial constraints, particularly in low-SES families, which could hinder their ability to pursue preemptive transplants despite the clear medical advantages. By contrast, living donors with fewer economic responsibilities, such as siblings or unrelated individuals, may reduce the correlation between SES and preemptive transplants. Our limited sample size precluded further stratification of living donors by relationship to the patient.

Although insurance type is a commonly used SES measure, insurance type did not emerge as a significant predictor of preemptive transplantation in our multivariable model. Our results indicate the superiority of the HOUSES index compared with insurance in predicting preemptive transplantation in children. In a systemic review of 29 studies, Pollack *et al.* noted a significant association between *wealth*, defined as assets and net worth, and health care outcomes after adjusting for common SES measures, including education level, income, and occupation.^21^ One could argue that HOUSES reflects wealth better than insurance type.

Racial disparities in preemptive transplantation have been reported previously. Patzer *et al.* reported that Black and Hispanic children were 66% and 52% less likely, respectively, compared with their White counterparts to receive a living donor preemptive transplant.^19^ Interestingly, in our cohort, race did not emerge as a significant predictor of preemptive transplant rates in either living or deceased donor recipients. This may be related to our small sample size and limited racial diversity. However, race did emerge as a significant predictor of prolonged dialysis duration in children who developed ESKD after 2 years.

Only 2.2% of the preemptive transplants in our study cohort were performed in children with FSGS as the underlying cause of kidney disease. This is not unexpected, as children with FSGS often undergo pretransplant bilateral nephrectomies to normalize serum albumin levels, reduce the thrombotic risk associated with nephrotic syndrome, and improve the ability to diagnose posttransplant FSGS recurrence by eliminating proteinuria from native kidneys.^22,23^ Pretransplant native nephrectomies necessitate dialysis initiation, precluding preemptive kidney transplantation.

SES may mediate its effect on preemptive transplants through several factors. Patients from disadvantaged backgrounds often encounter barriers, including limited access to nephrology care,^24^ delayed^25^ or no referral^26^ for transplant evaluation, and lack of awareness about transplant benefits. The latter is unsurprising as SES is intricately linked to health literacy.^27^ Since early referrals for transplant evaluation are associated with higher rates of preemptive transplants,^28^ the HOUSES index could be tested in the future as a measure to prompt earlier referral for those in the lower quartile and/or could be used to identify patients who might need more social work support earlier in the transplant process to help identify community resources. Increased attention and resources for lower SES patients may improve their access to timely transplants, setting the stage for successful long-term outcomes.

Our study has several limitations. Given the retrospective nature, we could not account for unknown confounders. Owing to a predominantly White patient population and a small sample size, we could not examine an interaction between HOUSES and race. HOUSES was computed based on addresses at the time of transplant (addresses at the time of dialysis initiation were unavailable). Hence, our pretransplant dialysis duration analysis was based on the assumption that SES was the same at the time of dialysis initiation as it was at the time of transplant. Owing to our limited sample size, we could not examine if the type of living donor affected the association between HOUSES and preemptive transplants. In addition, excluding 13 patients with missing addresses may have introduced selection bias by omitting recipients who may have faced housing insecurity. The results of our single-center study may not be generalizable to centers with different patient demographics. Furthermore, center-specific and state-related differences in policies and resources may amplify or diminish the relevance of socioeconomic challenges. As HOUSES is available nationwide, a multicenter national study should be conducted to study the effects of SES on transplant access, overcoming the limitations of our single-center study.

In summary, this study highlights significant socioeconomic disparities in preemptive kidney transplantation rates among children. The study also demonstrates the utility of HOUSES in uncovering socioeconomic disparities and identifying children at risk of nonpreemptive transplants. The findings underscore the need for targeted interventions to overcome socioeconomic barriers to timely transplantation. Early referral for transplant evaluation, timely listing, and resource allocation guided by HOUSES may be effective strategies to mitigate disparities. Future research should prioritize multicenter studies to clarify the effect of HOUSES on kidney transplant access and outcomes. In addition, investigations should examine the interactions between HOUSES and race as well as HOUSES and travel distance from transplant centers. Studies should also evaluate the effectiveness of HOUSES-based interventions in promoting preemptive listing and, ultimately, preemptive transplantation.

## Supplementary Material

## Data Availability

Partial restrictions to the data and/or materials apply. Data is available from the authors on request.

## Supplemental Material

Supplemental Table 1. Publications showing health disparities by HOUSES (adults; 39 outcomes).

Supplemental Table 2. Publications showing health disparities by HOUSES (children; 23 outcomes).

Supplemental Table 3. Preemptive transplantation and patient characteristics.

Supplemental Table 4. Pretransplant dialysis duration and patient characteristics.
