# Supplementary material for: Socioeconomic Disparities in Preemptive Kidney Transplant Rates in Children
Source: Kidney360. 2025 Apr 7;6(7):1188–97. doi: 10.34067/KID.0000000802 (PMC12338362; doi:10.34067/KID.0000000802)
Supplement: Supplementary file 1 [file kidney360-6-1188-s001.pdf]

ASN American Society of Nephrology | JASN Journal of the American Society of Nephrology | KIDNEY360 Accessing Our World From Every Angle | CJASN Clinical Journal of the American Society of Nephrology

ASN Journal Disclosure Form

As per ASN journal policy, I have disclosed any financial relationships or commitments I have held in the past 36 months as included below. I have listed my Current Employer below to indicate there is a relationship requiring disclosure. If no relationship exists, my Current Employer is not listed.

S. Amaral reports the following:
Employer: The Children's Hospital of Philadelphia; Consultancy: Bristol Myers Squibb - DSMB; CJASN Editorial Board; Otsuka- Advisory Board; NovoNordisk- Advisory Board; Research Funding: NIH- NIDDK, NICHD, NIAID; Laffey-McHugh Foundation; Honoraria: NIH Grant Reviewer; Advisory or Leadership Role: OPTN Board of Directors; and Other Interests or Relationships: ASPN Executive Council.

I understand that the information above will be published within the journal article, if accepted, and that failure to comply and/or to accurately and completely report the potential financial conflicts of interest could lead to the following: 1) Prior to publication, article rejection, or 2) Post-publication, sanctions ranging from, but not limited to, issuing a correction, reporting the inaccurate information to the authors' institution, banning authors from submitting work to ASN journals for varying lengths of time, and/or retraction of the published work.

Name: Sandra Amaral
Manuscript ID: K360-2025-000065R1
Manuscript Title: Socioeconomic Disparities in Preemptive Kidney Transplant Rates in Children
Date of Completion: March 14, 2025
Disclosure Updated Date: March 14, 2025

ASN
American Society of Nephrology
JASN
Journal of the American Society of Nephrology
KIDNEY360
Accessing Our World From Every Angle
CJASN
Clinical Journal of the American Society of Nephrology

ASN Journal Disclosure Form

As per ASN journal policy, I have disclosed any financial relationships or commitments I have held in the past 36 months as included below. I have listed my Current Employer below to indicate there is a relationship requiring disclosure. If no relationship exists, my Current Employer is not listed.

Y. Juhn reports the following:
Employer: Mayo Clinic; Research Funding: GSK; and Patents or Royalties: Patent 10654923; Pending Patent 630666.01468.

I understand that the information above will be published within the journal article, if accepted, and that failure to comply and/or to accurately and completely report the potential financial conflicts of interest could lead to the following: 1) Prior to publication, article rejection, or 2) Post-publication, sanctions ranging from, but not limited to, issuing a correction, reporting the inaccurate information to the authors' institution, banning authors from submitting work to ASN journals for varying lengths of time, and/or retraction of the published work.

Name: Young J Juhn
Manuscript ID: K360-2025-000065R1
Manuscript Title: Socioeconomic Disparities in Preemptive Kidney Transplant Rates in Children
Date of Completion: March 11, 2025
Disclosure Updated Date: March 11, 2025

ASN American Society of Nephrology | JASN Journal of the American Society of Nephrology | KIDNEY360 Accessing Our World From Every Angle | CJASN Clinical Journal of the American Society of Nephrology

ASN Journal Disclosure Form

As per ASN journal policy, I have disclosed any financial relationships or commitments I have held in the past 36 months as included below. I have listed my Current Employer below to indicate there is a relationship requiring disclosure. If no relationship exists, my Current Employer is not listed.

S. Kizilbash reports the following:
Employer: University of Minnesota; Mayo Clinic, Rochester; Consultancy: My spouse reports consulting agreements with SK Life Science and Apollomics, Inc. All consulting fees were paid to the institution. No personal compensation has been received for any of the abovementioned disclosures.; and Research Funding: My spouse reports grants from Orbus Therapeutics, Inc., Apollomics, Inc., Celgene, Wayshine Biopharma, Delmar Therapeutics, Inc., LOXO Oncology, Incyte, Nerviano Medical Sciences, all of which were paid to the institution and were used for clinical trial related expenses. I will receive research support from Eurofins Genomic. I am participating in a clinical trial sponsored by Akebia Therapeutics. I completed a research study sponsored by Diagnostic/Biotech Company, BioPorto.

I understand that the information above will be published within the journal article, if accepted, and that failure to comply and/or to accurately and completely report the potential financial conflicts of interest could lead to the following: 1) Prior to publication, article rejection, or 2) Post-publication, sanctions ranging from, but not limited to, issuing a correction, reporting the inaccurate information to the authors' institution, banning authors from submitting work to ASN journals for varying lengths of time, and/or retraction of the published work.

Name: Sarah J. Kizilbash
Manuscript ID: K360-2025-000065R1
Manuscript Title: Socioeconomic Disparities in Preemptive Kidney Transplant Rates in Children
Date of Completion: March 8, 2025
Disclosure Updated Date: May 21, 2024

ASN
American Society of Nephrology
JASN
Journal of the American Society of Nephrology
KIDNEY360
Accessing Our World From Every Angle
CJASN
Clinical Journal of the American Society of Nephrology

ASN Journal Disclosure Form

As per ASN journal policy, I have disclosed any financial relationships or commitments I have held in the past 36 months as included below. I have listed my Current Employer below to indicate there is a relationship requiring disclosure. If no relationship exists, my Current Employer is not listed.

W. Mckinney reports the following:
Employer: Hennepin Healthcare Research Institute; and Advisory or Leadership Role: I was appointed to the Board of Hennepin Healthcare Research Institute in Feb. 2022 after being promoted to Vice President of Equity in Research. The VP role is paid (via effort), but the Board role is not.

I understand that the information above will be published within the journal article, if accepted, and that failure to comply and/or to accurately and completely report the potential financial conflicts of interest could lead to the following: 1) Prior to publication, article rejection, or 2) Post-publication, sanctions ranging from, but not limited to, issuing a correction, reporting the inaccurate information to the authors' institution, banning authors from submitting work to ASN journals for varying lengths of time, and/or retraction of the published work.

Name: Warren T. Mckinney
Manuscript ID: K360-2025-000065R1
Manuscript Title: Socioeconomic Disparities in Preemptive Kidney Transplant Rates in Children
Date of Completion: March 10, 2025
Disclosure Updated Date: March 10, 2025

ASN American Society of Nephrology JASN Journal of the American Society of Nephrology KIDNEY360 Accessing Our World From Every Angle CJASN Clinical Journal of the American Society of Nephrology

ASN Journal Disclosure Form

As per ASN journal policy, I have disclosed any financial relationships or commitments I have held in the past 36 months as included below. I have listed my Current Employer below to indicate there is a relationship requiring disclosure. If no relationship exists, my Current Employer is not listed.

S. Riad reports the following:
Employer: Mayo Clinic

I understand that the information above will be published within the journal article, if accepted, and that failure to comply and/or to accurately and completely report the potential financial conflicts of interest could lead to the following: 1) Prior to publication, article rejection, or 2) Post-publication, sanctions ranging from, but not limited to, issuing a correction, reporting the inaccurate information to the authors' institution, banning authors from submitting work to ASN journals for varying lengths of time, and/or retraction of the published work.

Name: Samy M. Riad
Manuscript ID: K360-2025-000065R1
Manuscript Title: Socioeconomic Disparities in Preemptive Kidney Transplant Rates in Children
Date of Completion: March 11, 2025
Disclosure Updated Date: March 11, 2025

ASN
American Society of Nephrology
JASN
Journal of the American Society of Nephrology
KIDNEY360
Accessing Our World From Every Angle
CJASN
Clinical Journal of the American Society of Nephrology

ASN Journal Disclosure Form

As per ASN journal policy, I have disclosed any financial relationships or commitments I have held in the past 36 months as included below. I have listed my Current Employer below to indicate there is a relationship requiring disclosure. If no relationship exists, my Current Employer is not listed.

M. Roy reports the following:
Employer: Mayo Clinic

I understand that the information above will be published within the journal article, if accepted, and that failure to comply and/or to accurately and completely report the potential financial conflicts of interest could lead to the following: 1) Prior to publication, article rejection, or 2) Post-publication, sanctions ranging from, but not limited to, issuing a correction, reporting the inaccurate information to the authors' institution, banning authors from submitting work to ASN journals for varying lengths of time, and/or retraction of the published work.

Name: Madison Roy
Manuscript ID: K360-2025-000065R1
Manuscript Title: Socioeconomic Disparities in Preemptive Kidney Transplant Rates in Children
Date of Completion: March 20, 2025
Disclosure Updated Date: March 10, 2025

ASN
American Society of Nephrology
JASN
Journal of the American Society of Nephrology
KIDNEY360
Accessing Our World From Every Angle
CJASN
Clinical Journal of the American Society of Nephrology

ASN Journal Disclosure Form

As per ASN journal policy, I have disclosed any financial relationships or commitments I have held in the past 36 months as included below. I have listed my Current Employer below to indicate there is a relationship requiring disclosure. If no relationship exists, my Current Employer is not listed.

C. Wi has nothing to disclose.

I understand that the information above will be published within the journal article, if accepted, and that failure to comply and/or to accurately and completely report the potential financial conflicts of interest could lead to the following: 1) Prior to publication, article rejection, or 2) Post-publication, sanctions ranging from, but not limited to, issuing a correction, reporting the inaccurate information to the authors' institution, banning authors from submitting work to ASN journals for varying lengths of time, and/or retraction of the published work.

Name: Chung-Il Wi
Manuscript ID: K360-2025-000065R1
Manuscript Title: Socioeconomic Disparities in Preemptive Kidney Transplant Rates in Children
Date of Completion: March 27, 2025
Disclosure Updated Date: March 20, 2025
