# Supplementary material for: Socioeconomic Disparities in Preemptive Kidney Transplant Rates in Children
Source: Kidney360. 2025 Apr 7;6(7):1188–97. doi: 10.34067/KID.0000000802 (PMC12338362; doi:10.34067/KID.0000000802)
Supplement: Supplementary file 2 [file kidney360-6-1188-s002.pdf]

Supplemental Table 3: Preemptive Transplantation and Patient Characteristics

	**Non-preemptive transplant (N=127)**	**Preemptive transplant (N=46)**	**p-value**
**Age at ESKD**			0.19[1]
Median	11.3	11.7	
Range	0.01 - 17.9	0.96 - 17.9	
**Age at Transplant**			0.71[1]
Median	12.4	11.7	
Range	0.77 - 19.6	0.96 - 17.9	
**Sex**			0.007[2]
F	65 (51.2%)	13 (28.3%)	
M	62 (48.8%)	33 (71.7%)	
**Race/Ethnicity**			0.28[2]
N-Miss	7	2	
Non-Hispanic White	65 (54.2%)	28 (63.6%)	
Other	55 (45.8%)	16 (36.4%)	

**Donor Type**			0.15[2]
Deceased	57 (44.9%)	15 (32.6%)	
Living	70 (55.1%)	31 (67.4%)	
**Insurance Type**			0.003[2]
Private	68 (53.5%)	36 (78.3%)	
Public/Other	59 (46.5%)	10 (21.7%)	
**HOUSES Index**			0.024[2]
Q2-4	89 (70.1%)	40 (87.0%)	
Q1	38 (29.9%)	6 (13.0%)	
**Cause of ESKD**			0.004[2]
CAKUT	51 (40.2%)	31 (67.4%)	
FSGS	27 (21.3%)	1 (2.2%)	
Glomerulonephritis	6 (4.7%)	1 (2.2%)	
Other	33 (26.0%)	12 (26.1%)	
Unknown	10 (7.9%)	1 (2.2%)	

1 Linear Model ANOVA

2 Pearson's Chi-squared test

ESKD: end-stage kidney disease; CAKUT: congenital anomalies of the kidney and urinary tract; FSGS: focal segmental glomerular sclerosis

Supplement Table 4: Pretransplant Dialysis Duration and Patient Characteristics

	<1 year (N=63)	1+ years (N=37)	p-value
**Age at ESKD**			0.02[1]
Median	12.3	9.6	
Range	0.64 - 17.5	0.01 - 17.5	
Age at Transplant			0.43[1]
Median	12.7	12.3	
Range	1.1 - 17.9	1.1 - 19.6	
Sex			0.57[2]
F	36 (57.1%)	19 (51.4%)	
M	27 (42.9%)	18 (48.6%)	
Race/Ethnicity			0.001[2]
Non-Hispanic White	43 (68.3%)	13 (35.1%)	
Other	20 (31.7%)	24 (64.9%)	
Donor Type			< 0.001[2]
Deceased	22 (34.9%)	29 (78.4%)	
Living	41 (65.1%)	8 (21.6%)	
Insurance Type			< 0.001[2]
Private	43 (68.3%)	12 (32.4%)	
Public/Other	20 (31.7%)	25 (67.6%)	
Recipient BMI (at transplant)			0.77[1]
Median	18.2	18.8	
Range	12.2 - 36.9	14.3 - 36.7	

HOUSES Index			0.14[2]
Q 2-4	45 (71.4%)	21 (56.8%)	
Q1	18 (28.6%)	16 (43.2%)	
Cause of ESKD			0.76[2]
CAKUT	26 (41.3%)	11 (29.7%)	
FSGS	14 (22.2%)	9 (24.3%)	
Glomerulonephritis	3 (4.8%)	2 (5.4%)	
Other	14 (22.2%)	12 (32.4%)	
Unknown	6 (9.5%)	3 (8.1%)	

ESKD: end-stage kidney disease; CAKUT: congenital anomalies of the kidney and urinary tract; FSGS: focal segmental glomerular sclerosis
