# Supplementary material for: Socioeconomic Disparities in Preemptive Kidney Transplant Rates in Children
Source: Kidney360. 2025 Apr 7;6(7):1188–97. doi: 10.34067/KID.0000000802 (PMC12338362; doi:10.34067/KID.0000000802)
Supplement: Supplementary file 3 [file kidney360-6-1188-s003.pdf]

Supplemental Tables

Supplemental Table 1. Publications showing health disparities by HOUSES (adults) (39 outcomes)		
**Study**	**Outcomes**	**Adjusted effect size for Q1 (lowest SES by HOUSES index) (95% CI)[a]**
**1. Chronic conditions**		
Ahn, 2023[1]	**Alcohol-associated hepatitis**	Distribution of HOUSES: 39.9% (Q1), 12.5%% (Q2), 12.3% (Q3), and 17.1% (Q4)
Tadese, 2022[2]	**Severe obesity**	OR = 1.33 (0.99-1,80)
Greenwood, 2022[3]	**Diabetic control**	OR = 0.83 (0.70-0.98)
Rusk, 2022[4]	**Indigenous Smoking Behaviors**	Distribution of HOUSES in indigenous cohort (vs. match cohort): 39.9% (vs. 26.7%) for Q1, 12.5%% (vs. 24.3%) for Q2, 10.4% (vs. 20.6%) for Q3, and 8.0% (vs. 24.8%) for Q4
Rusk, 2022[5]	**Smoking Behaviors Among Indigenous Pregnant People**	Distribution of HOUSES in indigenous cohort (vs. match cohort): 40.4% (vs. 35.2%) for Q1, 33.3% (vs. 28.1%) for Q2, 12.3% (vs. 23.0%) for Q3, and 14.0% (vs. 13.8%) for Q4
Angstman, 2021[6]	**Persistent depressive symptoms**	OR = 1.31 (1.06-1.61)
	**Remission of depressive symptoms**	OR = 0.78 (0.65-0.95)
Thacher, 2020[7]	**25-Hydroxyvitamin D Level**	Estimate = 0.28 (0.21-0.35)
Stevens, 2020[8]	**Kidney transplantation failure**	HR = 2.0 (1.04-3.84)
Ryan, 2020[9]	**Post-Glioma mortality**	HR = 1.61 (1.05-2.5)
Patten, 2020[10]	**Mood disorder and obesity**	Distribution of HOUSES in rural (vs. urban): 11% (vs. 28%) for Q1, 21% (vs. 27%) for Q2, 28% (vs. 25%) for Q3, 40% (20%) for Q4
Takahashi, 2016[11]	**All-cause hospitalization**	HR = 1.53 (1.18-1.98)
	**Multiple chronic conditions prevalence**	OR = 2.4 (2.0-3.1)
Wi, 2016[12]	**Coronary heart disease prevalence**	OR = 1.35 (1.25-1.44)
	**Asthma prevalence**	OR = 1.28 (1.20-1.36)
	**Diabetes prevalence**	OR = 1.78 (1.66-1.92)
	**Hypertension prevalence**	OR = 1.56 (1.47-1.63)
	**Mood disorder prevalence**	OR = 1.63 (1.58-1.72)
Ghawi, 2015[13]	**Rheumatoid Arthritis incidence**	OR = 1.06 (1.02-1.09)
	**Post-Rheumatoid Arthritis mortality**	HR = 1.58 (1.05-2.36)
Bang, 2014[14]	**Post–Myocardial Infarction mortality**	HR = 1.86 (1.07-3.24)
**2. Acute conditions**		
Wi, 2023[15]	**Subject recruitment**	Distribution of HOUSES in Standard mailing (TESRS): 16.1% (vs. 12.9% for Q1, 9.7 (vs. 12.9%) for Q2, 45.2% (vs. 45.2%) for Q3, and 29.0% (vs. 29.0%) for Q4
Zurek, 2022[16]	**Hospital readmission**	OR* = 0.90 (0.83-0.98) for Q3
Vachon, 2022[17]	**COVID-19 asymptomatic rates**	HR* = 1.22 (0.86-1.7) for Q4
Juhn 2021[18]	**COVID-19 incidence in urban**	Identified geographical hotspots taking into account HOUSES

Wheeler 2021[19]	**COVID-19 incidence in rural**	Identified geographical hotspots taking into account HOUSES
Aul, 2020[20]	**Osteoporotic fracture incidence**	HR = 1.05 (1.04-1.08)
Barwise, 2020[21]	**Mortality rates (>50 years of age)**	HR = 1.38 (1.07-1.78)
Ryu, 2017[22]	**Accidental falls incidence**	HR = 1.72 (1.31-2.27)
**3. Behavioral risk factors and others**		
Felzer, 2023[23]	**Influenza vaccination in solid organ transplant patients**	RR* = 1.14 (1.008-1.30) for Q3
Juhn, 2021[24]	**Adherence to Public Health Measures Mitigates the Risk of COVID-19 Infection**	Used as a covariate for basic characteristics 12.3% (Q1), 25.5% (Q2), 28.3% (Q3), and 33.9% (Q4)
MacLaughlin, 2020[25]	**HPV Vaccination (initiation and completion)**	RR = 1.15 [1.03–1.28] for Q4 (initiation)<br>RR = 1.32 [1.21–1.44] for Q4 (completion)
Barwise, 2019[26]	**Advance Care Planning and Nursing Home Utilization**	OR* = 0.77 (0.63-0.93) for advance directives<br>OR* = 0.60 (0.50-0.72) for discharge to home
Ryu, 2018[27]	**Inconsistency of self-reported disease prevalence in survey results**	OR = 1.46 (1.17-1.84)
Barwise, 2018[28]	**Rates of social work consultation in end-of-life care**	OR = 1.46 (1.18-1.79)
	**Advance directives**	OR =1.29 (1.07-1.58)
Wi, 2016[29]	**Smoking status**	OR = 2.56 (1.14-5.55)
[a] Highest HOUSES group as a reference, except Zurek et al 2022* and Felzer et al 2022* which used lowest HOUSES group, Vachon et al 2022* which used 2nd quartile of HOUSES group, and Barwise et al 2019* which used 2nd-4th quartile of HOUSES group as a reference		

Supplemental Table 2. Publications showing health disparities by HOUSES (children) (23 outcomes)		
**Study**	**Outcomes**	**Adjusted effect size for Q1 (lowest SES by HOUSES index) (95% CI)[a]**
**1. Chronic conditions**		
Skolnick 2023[30]	**Severe Obesity**	OR = 2.78 (1.91-4.06)
Rodriguez, 2023[31]	**Acne prevalence**	Distribution of HOUSES in acne cases (vs. matched controls): 42.2% (vs. 32.7%) for Q1, 22.8% (vs. 25.5%) for Q2, 17.4% (vs. 20.1%) for Q3, and 17.5% (21.7%) for Q4
Bjur 2019[32]	**Asthma prevalence**	OR = 1.14 (1.03-1.29)
	**Epilepsy prevalence**	OR = 1.88 (1.21-2.85)
	**Mood disorders prevalence**	OR =1.38 (1.19-1.61)
Bjur 2019[33]	**Multiple complex chronic conditions prevalence**	5-year prevalence in 2004:<br>Q1 (1,443), Q2 (1,435), Q3 (1,134), Q4 (1,124)
Ryu 2016[34]	**Overweight prevalence**	OR = 2.08 (1.06-4) (OC) [b]<br>OR = 2.22 (1.12-4.34) (JC) [b]
	**Low birth weight prevalence**	OR = 1.28 (0.49-3.33) (OC) [b]<br>OR = 1.85 (0.82-2.94) (JC) [b]
	**Household smoking status**	OR = 4.16 (2.04-9.09) (OC) [b]<br>OR = 3.84 (2.27-6.25) (JC) [b]
Lynch 2015[35]	**Overweight/Obesity (HOUSES as covariate)**	Mean (SD) ($p<0.0001$)<br>Healthy weight: 1.7 (3.9)<br>Overweight: 1.0 (3.8)<br>Obesity: 0.1 (3.4)
Harris 2014[36]	**Poorly controlled asthma status**	OR = 4.76 (1.12-20)
Butterfield 2011[37]	**Adverse self-rated health**	OR = 4.76 (1.96-12.5) (OC) [b]<br>OR = 1.01 (0.24-4.0) (JC) [b]
Juhn 2011[38]	**Overweight prevalence**	OR=2.56 (p=0.008) (OC) [b]<br>OR=2.04 (p=0.07) (JC) [b]
	**Low birth weight prevalence**	OR=1.58 (p=0.330) (OC) [b]<br>OR=2.38 (p=0.018) (JC) [b]
	**Household smoking status prevalence**	OR=2.56 (p=0.007) (OC) [b]<br>OR=4.16 ($p<.001$) (JC) [b]
**2. Acute conditions**		
Patel 2020[22]	**Adverse childhood events risk (HOUSES as covariate)**	Proportion of outcomes by HOUSES quartile<br>Q1 (4.6%), Q2 (3.5%), Q3 (3.0%), Q4 (2.1%)
Bjur 2019[32]	**Bronchiolitis prevalence**	OR = 1.44 (1.28-1.63)
	**Pneumonia prevalence**	OR = 1.13 (1.01-1.28)
	**Urinary Tract Infection prevalence**	OR = 1.51 (1.28-1.78)
	**Adverse childhood experiences prevalence**	OR = 1.85 (1.51-2.27)
Johnson 2013[39]	**Invasive pneumococcal disease risk**	OR = 4.54 (1.12-20)
**3. Behavioral risk factors**		
Maclaughlin 2020[25]	**HPV vaccine initiation**	Rates: 0.86 (0.78-0.97)
	**HPV vaccine completion**	Rates: 0.75 (0.69-0.82)

Hammer 2016[40]	**Pertussis vaccine up-to-date status**	OR = 0.27 (0.10-0.74)
**4. AI bias**		
Juhn 2022[41]	**Performance of predicting risk of asthma exacerbation**	Balance error rate [(FPR+FNR)/2]<br>Q1 = 0.53, Q2-4: 0.39 (Ratio: 1.35)

[a] Highest HOUSES group as a reference; [b] OC: Olmsted county, Minnesota JC: Jackson county, Missouri

1. Ahn JC, Wi CI, Buryska S, et al. Disproportionate increases in alcohol-associated hepatitis incidence in women and individuals of low socioeconomic status: A population-based study using the Rochester epidemiology project database. Hepatology Communications 2023;7.
2. Tadese K, Ernst V, Weaver AL, et al. Association of Perinatal Factors With Severe Obesity and Dyslipidemia in Adulthood. J Prim Care Community Health 2022;13:21501327211058982.
3. Greenwood J, Zurek KI, Grimm JM, et al. Association of a housing based individual socioeconomic status measure with diabetic control in primary care practices. Prim Care Diabetes 2022;16:78-83.
4. Rusk AM, Giblon RE, Chamberlain AM, et al. Indigenous Smoking Behaviors in Olmsted County, Minnesota: A Longitudinal Population-Based Study. Mayo Clinic Proceedings 2022;97:1836-1848.
5. Rusk AM, Giblon RE, Chamberlain AM, et al. Smoking Behaviors Among Indigenous Pregnant People Compared to a Matched Regional Cohort. Nicotine & Tobacco Research 2023;25:889-897.
6. Angstman KB, Wi CI, Williams MD, et al. Impact of socioeconomic status on depression clinical outcomes at six months in a Midwestern, United States community. Journal of Affective Disorders 2021;292:751-756.
7. Thacher TD, Dudenkov DV, Mara KC, et al. The relationship of 25-hydroxyvitamin D concentrations and individual-level socioeconomic status. J Steroid Biochem Mol Biol 2020;197:105545.
8. Stevens MA, Beebe TJ, Wi C-I, et al. HOUSES index as an innovative socioeconomic measure predicts graft failure among kidney transplant recipients. Transplantation 2020;Online First.
9. Ryan CS, Juhn YJ, Kaur H, et al. Long-term incidence of glioma in Olmsted County, Minnesota, and disparities in postglioma survival rate: a population-based study. Neurooncol Pract 2020;7:288-298.
10. Patten CA, Juhn YJ, Ryu E, et al. Rural-urban health disparities for mood disorders and obesity in a midwestern community. Journal of Clinical and Translational Science 2020;4:408-415.
11. Takahashi PY, Ryu E, Hathcock MA, et al. A novel housing-based socioeconomic measure predicts hospitalisation and multiple chronic conditions in a community population. J Epidemiol Community Health 2016;70:286-91.
12. Wi CI, St Sauver JL, Jacobson DJ, et al. Ethnicity, Socioeconomic Status, and Health Disparities in a Mixed Rural-Urban US Community-Olmsted County, Minnesota. Mayo Clin Proc 2016;91:612-22.
13. Ghawi H, Crowson CS, Rand-Weaver J, et al. A novel measure of socioeconomic status using individual housing data to assess the association of SES with rheumatoid arthritis and its mortality: a population-based case-control study. BMJ Open 2015;5:e006469.
14. Bang DW, Manemann SM, Gerber Y, et al. A novel socioeconomic measure using individual housing data in cardiovascular outcome research. Int J Environ Res Public Health 2014;11:11597-615.
15. Wi CI, King KS, Ryu E, et al. Application of Innovative Subject Recruitment System for Batch Enrollment: A Pilot Study. Journal of Primary Care and Community Health 2023;14.
16. Zurek KI, Boswell CL, N EM, et al. Association of Early and Late Hospital Readmissions with a Novel Housing-Based Socioeconomic Measure. Health Serv Res Manag Epidemiol 2022;9:23333928221104644.
17. Vachon CM, Norman AD, Prasad K, et al. Rates of Asymptomatic COVID-19 Infection and Associated Factors in Olmsted County, Minnesota, in the Prevaccination Era. Mayo Clin Proc Innov Qual Outcomes 2022;6:605-617.

18. Juhn YJ, Wheeler P, Wi CI, et al. Role of Geographic Risk Factors in COVID-19 Epidemiology: Longitudinal Geospatial Analysis. Mayo Clin Proc Innov Qual Outcomes 2021;5:916-927.
19. Wheeler PH, Patten CA, Wi CI, et al. Role of geographic risk factors and social determinants of health in COVID-19 epidemiology: Longitudinal geospatial analysis in a midwest rural region. Journal of Clinical and Translational Science 2021;6.
20. Aul AJ, Dudenkov DV, Mara KC, et al. The relationship of 25-hydroxyvitamin D values and risk of fracture: a population-based retrospective cohort study. Osteoporos Int 2020;31:1787-1799.
21. Barwise A, Wi CI, Frank R, et al. An Innovative Individual-Level Socioeconomic Measure Predicts Critical Care Outcomes in Older Adults: A Population-Based Study. J Intensive Care Med 2020:885066620931020.
22. Ryu E, Juhn YJ, Wheeler PH, et al. Individual housing-based socioeconomic status predicts risk of accidental falls among adults. Ann Epidemiol 2017;27:415-420.e2.
23. Felzer JR, Rutten LFJ, Wi CI, et al. Disparities in vaccination rates in solid organ transplant patients. Transplant Infectious Disease 2023;25.
24. Juhn YJ, Wi CI, Ryu E, et al. Adherence to Public Health Measures Mitigates the Risk of COVID-19 Infection in Older Adults: A Community-Based Study. Mayo Clin Proc 2021;96:912-920.
25. MacLaughlin KL, Jacobson RM, Sauver JLS, et al. An innovative housing-related measure for individual socioeconomic status and human papillomavirus vaccination coverage: A population-based cross-sectional study. Vaccine 2020;38:6112-6119.
26. Barwise A, Juhn YJ, Wi CI, et al. An Individual Housing-Based Socioeconomic Status Measure Predicts Advance Care Planning and Nursing Home Utilization. American Journal of Hospice & Palliative Medicine 2019;36:362-369.
27. Ryu E, Olson JE, Juhn YJ, et al. Association between an individual housing-based socioeconomic index and inconsistent self-reporting of health conditions: a prospective cohort study in the Mayo Clinic Biobank. BMJ Open 2018;8:e020054.
28. Barwise A, Juhn YJ, Wi CI, et al. An Individual Housing-Based Socioeconomic Status Measure Predicts Advance Care Planning and Nursing Home Utilization. Am J Hosp Palliat Care 2018.
29. Wi CI, Gauger J, Bachman M, et al. Role of individual-housing-based socioeconomic status measure in relation to smoking status among late adolescents with asthma. Ann Epidemiol 2016;26:455-60.
30. Skolnick V, Rajjo T, Thacher T, et al. Association of Weight Trajectory with Severe Obesity: A Case-Control Study. Childhood Obesity 2023.
31. Baisi KRE, Weaver AL, Wi CI, et al. Socioeconomic status, race, and preadolescent acne: A population-based retrospective cohort analysis in a mixed rural-urban community of the United States (Olmsted County, Minnesota). Pediatric Dermatology 2023;40:460-465.
32. Bjur KA, Wi CI, Ryu E, et al. Socioeconomic Status, Race/Ethnicity, and Health Disparities in Children and Adolescents in a Mixed Rural-Urban Community-Olmsted County, Minnesota. Mayo Clin Proc 2019;94:44-53.
33. Bjur KA, Wi CI, Ryu E, et al. Epidemiology of Children With Multiple Complex Chronic Conditions in a Mixed Urban-Rural US Community. Hosp Pediatr 2019;9:281-290.
34. Ryu E, Wi CI, Crow SS, et al. Assessing health disparities in children using a modified housing-related socioeconomic status measure: a cross-sectional study. BMJ Open 2016;6:e011564.
35. Lynch BA, Finney Rutten LJ, Jacobson RM, et al. Health Care Utilization by Body Mass Index in a Pediatric Population. Acad Pediatr 2015;15:644-50.
36. Harris MN, Lundien MC, Finnie DM, et al. Application of a novel socioeconomic measure using individual housing data in asthma research: an exploratory study. NPJ primary care respiratory medicine 2014;24:14018.

37. Butterfield MC, Williams AR, Beebe T, et al. A two-county comparison of the HOUSES index on predicting self-rated health. J Epidemiol Community Health 2011;65.
38. Juhn YJ, Beebe TJ, Finnie DM, et al. Development and initial testing of a new socioeconomic status measure based on housing data. J Urban Health 2011;88.
39. Johnson MD, Urm SH, Jung JA, et al. Housing data-based socioeconomic index and risk of invasive pneumococcal disease: an exploratory study. Epidemiology and infection 2013;141:880-7.
40. Hammer R, Capili C, Wi C-I, et al. A new socioeconomic status measure for vaccine research in children using individual housing data: a population-based case-control study. BMC Public Health 2016;16:1-9.
41. Juhn YJ, Ryu E, Wi CI, et al. Assessing socioeconomic bias in machine learning algorithms in health care: a case study of the HOUSES index. Journal of the American Medical Informatics Association 2022;29:1142-1151.
